# Tris{2-[(3-thien­yl)methyl­idene­amino]­eth­yl}amine

**DOI:** 10.1107/S1600536810039206

**Published:** 2010-10-09

**Authors:** Muhammet Işıklan, Avijit Pramanik, Frank R. Fronczek, Md. Alamgir Hossain

**Affiliations:** a Department of Chemistry and Biochemistry, Jackson State University, Jackson, MS 39217, USA; b Department of Chemistry, Louisiana State University, Baton Rouge, LA 70803, USA

## Abstract

The title compound, C_21_H_24_N_4_S_3_, is a tripodal Schiff base that was obtained from the reaction of tris­(2-amino­eth­yl)amine (tren) and thio­phene-3-carbaldehyde. The compound forms a cavity with approximate *C*3 symmetry. One of the thio­phene units is disordered in a 0.764 (2):0.236 (2) ratio. In the crystal, the three thio­phene ligands are involved in intra­molecular C—H⋯π inter­actions and the mol­ecules are connected by C—H⋯N inter­actions, forming hydrogen-bonded chains.

## Related literature

For general background to tren-based imines, see: Ballester *et al.* (1999 ▶); Bianchi *et al.* (1997 ▶); Fan *et al.* (2002 ▶); Kang *et al.* (2005 ▶); McLachlan *et al.* (1996 ▶); Kaur *et al.* (2009 ▶); Salehzadeh *et al.* (2006 ▶). For related structures, see: Alyea *et al.* (1989 ▶); Bazzicalupi *et al.* (2009 ▶); Burgess *et al.* (1991 ▶); Hossain *et al.* (2004 ▶); Mazik *et al.* (2001 ▶).
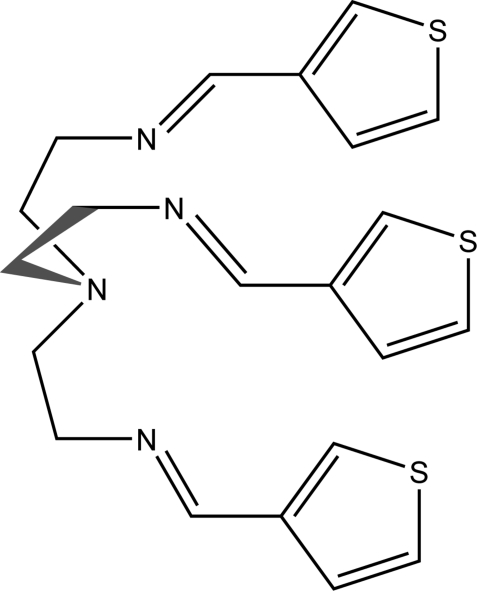


## Experimental

### 

#### Crystal data

C_21_H_24_N_4_S_3_
*M* *_r_* = 428.62
Monoclinic, 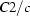

*a* = 28.694 (3) Å
*b* = 9.2529 (10) Å
*c* = 16.427 (2) Å
β = 96.150 (5)°
*V* = 4336.3 (8) Å^3^
*Z* = 8
Cu *K*α radiation
μ = 3.23 mm^−1^
*T* = 90 K
0.30 × 0.28 × 0.22 mm

#### Data collection

Bruker APEXII CCD diffractometer
Absorption correction: multi-scan (*SADABS*; Sheldrick, 2004 ▶) *T*_min_ = 0.444, *T*_max_ = 0.537
13414 measured reflections
3884 independent reflections
3571 reflections with *I* > 2σ(*I*)
*R*_int_ = 0.031

#### Refinement

*R*[*F* ^2^ > 2σ(*F* ^2^)] = 0.035
*wR*(*F* ^2^) = 0.092
*S* = 1.04
3884 reflections
261 parameters
28 restraints
H-atom parameters constrained
Δρ_max_ = 0.41 e Å^−3^
Δρ_min_ = −0.44 e Å^−3^

### 

Data collection: *APEX2* (Bruker, 2006 ▶); cell refinement: *SAINT* (Bruker, 2006 ▶); data reduction: *SAINT*; program(s) used to solve structure: *SHELXS97* (Sheldrick, 2008 ▶); program(s) used to refine structure: *SHELXL97* (Sheldrick, 2008 ▶); molecular graphics: *ORTEP-3 for Windows* (Farrugia, 1997 ▶); software used to prepare material for publication: *SHELXTL* (Sheldrick, 2008 ▶).

## Supplementary Material

Crystal structure: contains datablocks global, I. DOI: 10.1107/S1600536810039206/zq2062sup1.cif

Structure factors: contains datablocks I. DOI: 10.1107/S1600536810039206/zq2062Isup2.hkl

Additional supplementary materials:  crystallographic information; 3D view; checkCIF report

## Figures and Tables

**Table 1:** Hydrogen-bond geometry (Å, °) *Cg*1, *Cg*2 and *Cg*3 are the centroids of the S1,C4–C7, S2,C11–C14 and S3,C18–C21 rings, respectively.

*D*—H⋯*A*	*D*—H	H⋯*A*	*D*⋯*A*	*D*—H⋯*A*
C20—H20⋯N2^i^	0.95	2.55	3.354 (7)	143
C21—H21⋯*Cg*1	0.95	2.61	3.437 (2)	146
C5—H5⋯*Cg*2	0.95	2.65	3.452 (2)	142
C12—H12⋯*Cg*3	0.95	2.61	3.432 (3)	145

Symmetry code: (i) 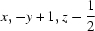.

**Fig. 1.**
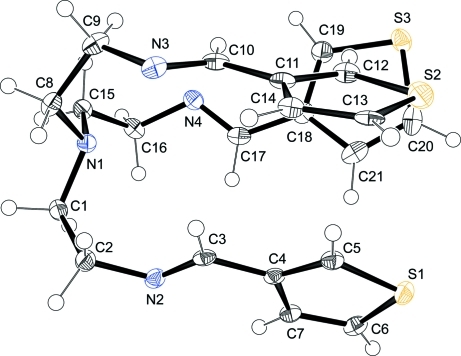
The structure of the title compound, with displacement ellipsoids drawn at the 50% probability level and H atoms with arbitrary radius.

**Fig. 2.**
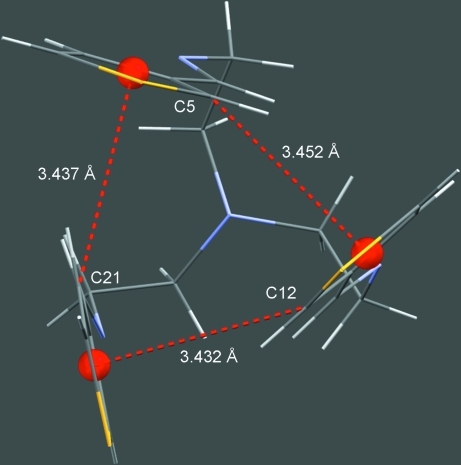
CH···π interactions (dotted lines) in the crystal structure of the title compound.

**Fig. 3.**
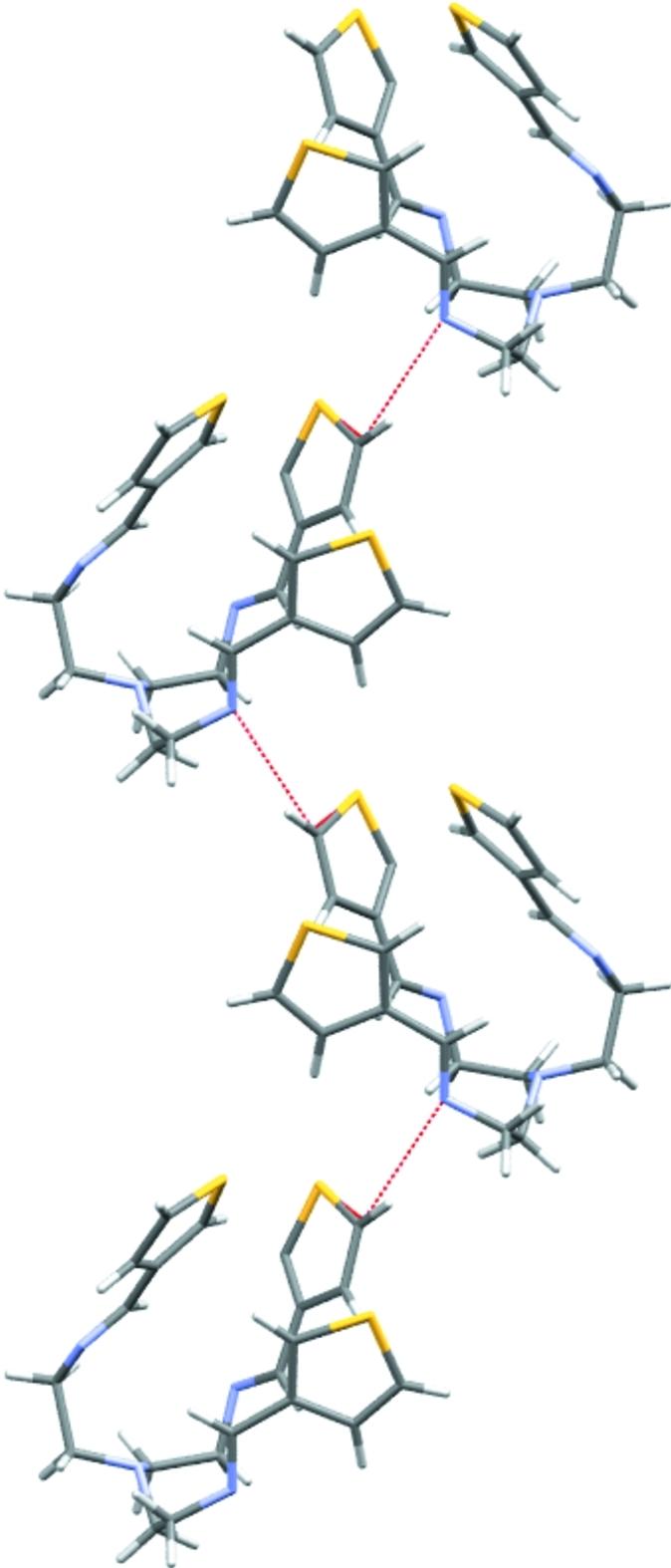
Hydrogen bonded chain in the lattice of the title compound viewed down a axis.

## supplementary crystallographic information

### Comment

Tren-based imines synthesized from the reaction of tris(2-aminoethyl)amine (tren) and an aldehyde are versatile ligands for transitional metals (Salehzadeh *et al.*, 2006; McLachlan *et al.*, 1996). Because of the simplicity, such imines are often converted directly into the corresponding amines, which are potential to bind a wide range of cations and anions (Bianchi *et al.*, 1997; Kang *et al.*, 2005). The connecting arms play an important role in achieving shape and size selectivity of a particular guest. Compared with a monopodal or dipodal receptor, a tripodal receptor often binds a guest species strongly due to the enhanced chelating effect and controlled cavity (Ballester *et al.*, 1999; Fan *et al.*, 2002). Therefore, an increasing attention is being paid to the development of new tripodal receptors (Kaur *et al.*, 2009). During the course of our study, we synthesized a new Schiff base from the reaction of tris(2-aminoethyl)amine (tren) and 3-thiophene aldehyde, and obtained crystals. Herein, we report the structure of tris[(4–2-thienyl)-3-aza-3-butenyl]amine (I), which was prepared by condensation of 3-thiophene aldehyde with tris(2-aminoethyl)amine. A related Schiff base, tris[4-(2-thienyl)-3-aza-3-butenyl]amine was synthesized and analyzed previously by crystallography (Alyea *et al.*, 1989). Although our compound is isomerically different, it shows almost similar cell parameters observed in the tris[(4–2-thienyl)-3-aza-3-butenyl]amine.

The structural analysis of the title compound shows that it forms a cavity with three arms (Figure 1). The compound contains an approximate *C_3_* symmetry axis passing through the tertiary N atom. One of the thiophene moieties is disordered by twofold rotation about C17—C18. All three aromatic units are involved in CH···π interactions with C···centroid distances of 3.452 (2), 3.432 (2) and 3.437 (3) Å (Table 1 and Figure 2). A related tren based receptor with three phenyl groups was reported earlier, showing a relatively flat structure where no CH···π interaction was observed (Hossain *et al.*, 2004). The presence of sulfur in the aromatic rings perhaps facilitates CH···π interactions by increasing the electron density to aromatic rings. In the crystal structure, neighboring units are connected by intermolecular C20—H20···N2 interactions (C···N = 3.354 (7) Å), forming hydrogen-bonded chains (Figure 3). Such distances are comparable with those observed in the crystal structure of an α,β-unsaturated ketone (CH···N interactions with C···N = 3.41 to 3.71 Å) with a terminal pyridine subunit (Mazik *et al.*, 2001).

### Experimental

To a solution of 3-thiophene aldehyde (2.30 g, 20.5 mmol) in diethylether (50 ml) was added tris(2-aminoethyl)amine (1.00 g, 6.84 mmol) in ethanol (50 ml). The mixture was stirred overnight at room temperature, and the solvent was evaporated. The product was suspended in water (50 ml) and an extraction was made with CH_2_Cl_2_(3 *x* 50 ml). The organic layers were combined and dried by anhydrous MgSO_4_ (1.5 g). The yellowish solution was collected by filtration, and the solvent was evaporated under reduced pressure. The crude product was purified by column chromatography on a neutral-alumina column (2% CH_3_OH in CH_2_Cl_2_) to give the imine as a white powder. Yield = 3.98 g (67%). M.P. 80 °C. ^1^H NMR (500 MHz, CDCl_3_, TMS): δ 2.85 (t, 6H, NC*H*_2_),3.59 ((t, 6H, NCH_2_C*H*_2_), δ 8.011 (s, 3H, NC*H*), 7.11 (m, 3H, Ar*H*), 7.28 (m, 3H, Ar*H)*, 7.45 (m, 3H, Ar*H*). The compound was redissolved in ethanol (1 ml) and crystals suitable for X-ray analysis were grown from slow evaporation of the solvent at room temperature.

### Refinement

H atoms on C were placed in idealized positions with C—H distances 0.95 - 0.99 Å and thereafter treated as riding. *U*_iso_ for H were assigned as 1.2 times *U*_eq_ of the attached C atom. The disorder in the thiophene ring containing S3 was modeled with two orientations having populations 0.764 (2) and 0.236 (2), their geometries being restrained to be the same as that of the thiophene containing S1. Displacement parameters of S3 and S3A were constrained to be equal, as were those of C20 and C20A.

### Crystal data

**Table:**

C_21_H_24_N_4_S_3_	*F*(000) = 1808
*M**_r_* = 428.62	*D*_x_ = 1.313 Mg m^−^^3^
Monoclinic, *C*2/*c*	Cu *K*α radiation, λ = 1.54178 Å
Hall symbol: -C 2yc	Cell parameters from 7541 reflections
*a* = 28.694 (3) Å	θ = 5.0–68.3°
*b* = 9.2529 (10) Å	µ = 3.23 mm^−^^1^
*c* = 16.427 (2) Å	*T* = 90 K
β = 96.150 (5)°	Fragment, colourless
*V* = 4336.3 (8) Å^3^	0.30 × 0.28 × 0.22 mm
*Z* = 8	

### Data collection

**Table:**

Bruker APEXII CCD diffractometer	3884 independent reflections
Radiation source: fine-focus sealed tube	3571 reflections with *I* > 2σ(*I*)
graphite	*R*_int_ = 0.031
φ and ω scans	θ_max_ = 68.3°, θ_min_ = 3.1°
Absorption correction: multi-scan (*SADABS*; Sheldrick, 2004)	*h* = −34→33
*T*_min_ = 0.444, *T*_max_ = 0.537	*k* = −11→10
13414 measured reflections	*l* = −19→17

### Refinement

**Table:**

Refinement on *F*^2^	Secondary atom site location: difference Fourier map
Least-squares matrix: full	Hydrogen site location: inferred from neighbouring sites
*R*[*F*^2^ > 2σ(*F*^2^)] = 0.035	H-atom parameters constrained
*wR*(*F*^2^) = 0.092	*w* = 1/[σ^2^(*F*_o_^2^) + (0.0403*P*)^2^ + 7.6616*P*] where *P* = (*F*_o_^2^ + 2*F*_c_^2^)/3
*S* = 1.03	(Δ/σ)_max_ < 0.001
3884 reflections	Δρ_max_ = 0.41 e Å^−^^3^
261 parameters	Δρ_min_ = −0.44 e Å^−^^3^
28 restraints	Extinction correction: *SHELXL97* (Sheldrick, 2008), Fc^*^=kFc[1+0.001xFc^2^λ^3^/sin(2θ)]^-1/4^
Primary atom site location: structure-invariant direct methods	Extinction coefficient: 0.00060 (5)

### Special details

**Table:**

Geometry. All e.s.d.'s (except the e.s.d. in the dihedral angle between two l.s. planes) are estimated using the full covariance matrix. The cell e.s.d.'s are taken into account individually in the estimation of e.s.d.'s in distances, angles and torsion angles; correlations between e.s.d.'s in cell parameters are only used when they are defined by crystal symmetry. An approximate (isotropic) treatment of cell e.s.d.'s is used for estimating e.s.d.'s involving l.s. planes.
Refinement. Refinement of *F*^2^ against ALL reflections. The weighted *R*-factor *wR* and goodness of fit *S* are based on *F*^2^, conventional *R*-factors *R* are based on *F*, with *F* set to zero for negative *F*^2^. The threshold expression of *F*^2^ > σ(*F*^2^) is used only for calculating *R*-factors(gt) *etc*. and is not relevant to the choice of reflections for refinement. *R*-factors based on *F*^2^ are statistically about twice as large as those based on *F*, and *R*- factors based on ALL data will be even larger.

### Fractional atomic coordinates and isotropic or equivalent isotropic displacement parameters (Å^2^)

**Table:**

	*x*	*y*	*z*	*U*_iso_*/*U*_eq_	Occ. (<1)
S2	0.292078 (17)	0.23863 (5)	0.25479 (3)	0.02483 (15)	
N1	0.42630 (5)	0.04730 (16)	0.62236 (9)	0.0153 (3)	
N2	0.34778 (5)	0.26041 (17)	0.65167 (9)	0.0174 (3)	
N3	0.37162 (5)	−0.07966 (17)	0.46740 (9)	0.0188 (3)	
N4	0.47792 (5)	0.26700 (17)	0.52115 (9)	0.0179 (3)	
C1	0.40953 (6)	0.0885 (2)	0.70049 (11)	0.0180 (4)	
H1A	0.4269	0.1750	0.7224	0.022*	
H1B	0.4162	0.0090	0.7404	0.022*	
C2	0.35725 (6)	0.1209 (2)	0.69159 (11)	0.0198 (4)	
H2A	0.3402	0.0440	0.6588	0.024*	
H2B	0.3458	0.1222	0.7463	0.024*	
C3	0.32751 (6)	0.2597 (2)	0.57929 (11)	0.0155 (4)	
H3	0.3206	0.1700	0.5525	0.019*	
S1	0.276626 (16)	0.57210 (5)	0.42956 (3)	0.02137 (14)	
C4	0.31449 (6)	0.3943 (2)	0.53614 (11)	0.0148 (4)	
C5	0.28869 (6)	0.3986 (2)	0.46128 (11)	0.0175 (4)	
H5	0.2786	0.3153	0.4305	0.021*	
C6	0.30664 (6)	0.6422 (2)	0.51705 (11)	0.0194 (4)	
H6	0.3100	0.7425	0.5283	0.023*	
C7	0.32484 (6)	0.5360 (2)	0.56788 (11)	0.0166 (4)	
H7	0.3426	0.5539	0.6191	0.020*	
C8	0.41542 (6)	−0.1043 (2)	0.60370 (12)	0.0191 (4)	
H8A	0.3847	−0.1282	0.6224	0.023*	
H8B	0.4394	−0.1662	0.6343	0.023*	
C9	0.41392 (6)	−0.1375 (2)	0.51287 (12)	0.0212 (4)	
H9A	0.4418	−0.0951	0.4913	0.025*	
H9B	0.4150	−0.2435	0.5049	0.025*	
C10	0.37694 (6)	0.0228 (2)	0.41808 (11)	0.0171 (4)	
H10	0.4075	0.0603	0.4150	0.021*	
C11	0.33762 (6)	0.0855 (2)	0.36569 (11)	0.0165 (4)	
C12	0.34322 (6)	0.1941 (2)	0.31106 (11)	0.0195 (4)	
H12	0.3724	0.2395	0.3052	0.023*	
C13	0.26031 (6)	0.11281 (19)	0.30122 (11)	0.0156 (4)	
H13	0.2277	0.0957	0.2886	0.019*	
C14	0.29004 (6)	0.0380 (2)	0.36131 (11)	0.0174 (4)	
H14	0.2795	−0.0358	0.3951	0.021*	
C15	0.47680 (6)	0.0740 (2)	0.62364 (11)	0.0172 (4)	
H15A	0.4893	0.0128	0.5816	0.021*	
H15B	0.4926	0.0450	0.6777	0.021*	
C16	0.48832 (6)	0.2312 (2)	0.60779 (11)	0.0194 (4)	
H16A	0.4698	0.2945	0.6407	0.023*	
H16B	0.5220	0.2490	0.6251	0.023*	
C17	0.44833 (6)	0.3661 (2)	0.50276 (11)	0.0179 (4)	
H17	0.4334	0.4100	0.5453	0.021*	
S3	0.43695 (3)	0.45750 (11)	0.26243 (4)	0.0223 (3)	0.764 (2)
C20	0.3950 (3)	0.5473 (10)	0.3129 (5)	0.0236 (13)	0.764 (2)
H20	0.3724	0.6120	0.2871	0.028*	0.764 (2)
S3A	0.3904 (3)	0.5578 (10)	0.3071 (4)	0.0223 (3)	0.236 (2)
C20A	0.4402 (5)	0.4535 (15)	0.2850 (7)	0.0236 (13)	0.236 (2)
H20A	0.4517	0.4506	0.2329	0.028*	0.236 (2)
C18	0.43616 (6)	0.4160 (2)	0.41825 (11)	0.0178 (4)	
C19	0.45957 (6)	0.3789 (2)	0.35179 (11)	0.0215 (4)	
H19	0.4858	0.3156	0.3557	0.026*	0.764 (2)
H19A	0.4849	0.3126	0.3526	0.026*	0.236 (2)
C21	0.39888 (6)	0.5126 (2)	0.39551 (13)	0.0230 (4)	
H21	0.3787	0.5496	0.4327	0.028*	0.764 (2)
H21A	0.3797	0.5479	0.4348	0.028*	0.236 (2)

### Atomic displacement parameters (Å^2^)

**Table:**

	*U*^11^	*U*^22^	*U*^33^	*U*^12^	*U*^13^	*U*^23^
S2	0.0283 (3)	0.0242 (3)	0.0219 (3)	−0.00055 (19)	0.00263 (19)	0.00012 (19)
N1	0.0126 (7)	0.0167 (8)	0.0168 (7)	0.0004 (6)	0.0022 (6)	0.0013 (6)
N2	0.0138 (7)	0.0177 (8)	0.0212 (8)	0.0023 (6)	0.0038 (6)	0.0021 (6)
N3	0.0157 (8)	0.0177 (8)	0.0229 (8)	−0.0007 (6)	0.0014 (6)	−0.0038 (7)
N4	0.0161 (7)	0.0199 (8)	0.0182 (8)	−0.0030 (6)	0.0036 (6)	0.0007 (6)
C1	0.0186 (9)	0.0199 (9)	0.0157 (8)	0.0021 (7)	0.0029 (7)	0.0037 (7)
C2	0.0183 (9)	0.0195 (10)	0.0225 (9)	0.0025 (7)	0.0061 (7)	0.0052 (8)
C3	0.0115 (8)	0.0150 (9)	0.0211 (9)	0.0000 (7)	0.0067 (7)	−0.0020 (7)
S1	0.0178 (2)	0.0234 (3)	0.0225 (2)	0.00528 (18)	0.00034 (17)	0.00436 (18)
C4	0.0096 (8)	0.0174 (9)	0.0181 (8)	0.0010 (7)	0.0054 (6)	−0.0002 (7)
C5	0.0142 (8)	0.0198 (9)	0.0190 (9)	0.0008 (7)	0.0038 (7)	−0.0018 (7)
C6	0.0167 (9)	0.0144 (9)	0.0277 (10)	0.0004 (7)	0.0057 (7)	−0.0012 (8)
C7	0.0131 (8)	0.0175 (9)	0.0194 (9)	0.0006 (7)	0.0032 (7)	−0.0019 (7)
C8	0.0161 (9)	0.0159 (9)	0.0252 (9)	0.0014 (7)	0.0011 (7)	0.0035 (8)
C9	0.0162 (9)	0.0176 (10)	0.0293 (10)	0.0020 (7)	0.0004 (7)	−0.0048 (8)
C10	0.0129 (8)	0.0188 (10)	0.0205 (9)	−0.0033 (7)	0.0057 (7)	−0.0082 (8)
C11	0.0161 (9)	0.0166 (9)	0.0174 (9)	−0.0016 (7)	0.0050 (7)	−0.0070 (7)
C12	0.0190 (9)	0.0182 (9)	0.0223 (9)	−0.0039 (7)	0.0067 (7)	−0.0048 (8)
C13	0.0141 (8)	0.0148 (9)	0.0198 (9)	−0.0054 (7)	0.0109 (7)	−0.0080 (7)
C14	0.0166 (9)	0.0184 (9)	0.0179 (9)	−0.0018 (7)	0.0045 (7)	−0.0038 (7)
C15	0.0124 (9)	0.0228 (10)	0.0160 (8)	0.0011 (7)	−0.0001 (7)	0.0014 (7)
C16	0.0170 (9)	0.0250 (10)	0.0163 (8)	−0.0042 (7)	0.0018 (7)	−0.0009 (7)
C17	0.0143 (8)	0.0188 (9)	0.0212 (9)	−0.0045 (7)	0.0051 (7)	−0.0053 (7)
S3	0.0200 (4)	0.0282 (4)	0.0190 (4)	−0.0019 (3)	0.0040 (3)	0.0048 (3)
C20	0.023 (3)	0.017 (2)	0.028 (2)	−0.0090 (16)	−0.0093 (16)	0.0049 (15)
S3A	0.0200 (4)	0.0282 (4)	0.0190 (4)	−0.0019 (3)	0.0040 (3)	0.0048 (3)
C20A	0.023 (3)	0.017 (2)	0.028 (2)	−0.0090 (16)	−0.0093 (16)	0.0049 (15)
C18	0.0131 (8)	0.0159 (9)	0.0246 (9)	−0.0044 (7)	0.0026 (7)	−0.0002 (7)
C19	0.0172 (9)	0.0246 (10)	0.0232 (9)	−0.0010 (8)	0.0052 (7)	0.0027 (8)
C21	0.0149 (9)	0.0166 (10)	0.0374 (11)	−0.0029 (7)	0.0028 (8)	−0.0044 (8)

### Geometric parameters (Å, °)

**Table:**

S2—C12	1.6990 (19)	C9—H9B	0.9900
S2—C13	1.7079 (18)	C10—C11	1.464 (3)
N1—C8	1.462 (2)	C10—H10	0.9500
N1—C15	1.468 (2)	C11—C12	1.368 (3)
N1—C1	1.468 (2)	C11—C14	1.429 (3)
N2—C3	1.266 (2)	C12—H12	0.9500
N2—C2	1.460 (2)	C13—C14	1.414 (3)
N3—C10	1.267 (2)	C13—H13	0.9500
N3—C9	1.457 (2)	C14—H14	0.9500
N4—C17	1.264 (2)	C15—C16	1.521 (3)
N4—C16	1.461 (2)	C15—H15A	0.9900
C1—C2	1.522 (2)	C15—H15B	0.9900
C1—H1A	0.9900	C16—H16A	0.9900
C1—H1B	0.9900	C16—H16B	0.9900
C2—H2A	0.9900	C17—C18	1.469 (3)
C2—H2B	0.9900	C17—H17	0.9500
C3—C4	1.461 (2)	S3—C19	1.703 (2)
C3—H3	0.9500	S3—C20	1.743 (7)
S1—C5	1.7113 (19)	C20—C21	1.387 (8)
S1—C6	1.7211 (19)	C20—H20	0.9500
C4—C5	1.367 (3)	S3A—C21	1.506 (7)
C4—C7	1.431 (3)	S3A—C20A	1.793 (13)
C5—H5	0.9500	C20A—C19	1.363 (11)
C6—C7	1.357 (3)	C20A—H20A	0.9500
C6—H6	0.9500	C18—C19	1.385 (3)
C7—H7	0.9500	C18—C21	1.413 (3)
C8—C9	1.520 (3)	C19—H19	0.9500
C8—H8A	0.9900	C19—H19A	0.9500
C8—H8B	0.9900	C21—H21	0.9500
C9—H9A	0.9900	C21—H21A	0.9500
			
C12—S2—C13	93.62 (9)	C14—C11—C10	125.46 (17)
C8—N1—C15	110.65 (14)	C11—C12—S2	112.30 (14)
C8—N1—C1	110.47 (14)	C11—C12—H12	123.9
C15—N1—C1	111.05 (14)	S2—C12—H12	123.9
C3—N2—C2	117.52 (16)	C14—C13—S2	109.53 (13)
C10—N3—C9	116.86 (16)	C14—C13—H13	125.2
C17—N4—C16	117.33 (16)	S2—C13—H13	125.2
N1—C1—C2	112.29 (14)	C13—C14—C11	112.72 (16)
N1—C1—H1A	109.1	C13—C14—H14	123.6
C2—C1—H1A	109.1	C11—C14—H14	123.6
N1—C1—H1B	109.1	N1—C15—C16	113.01 (15)
C2—C1—H1B	109.1	N1—C15—H15A	109.0
H1A—C1—H1B	107.9	C16—C15—H15A	109.0
N2—C2—C1	110.59 (15)	N1—C15—H15B	109.0
N2—C2—H2A	109.5	C16—C15—H15B	109.0
C1—C2—H2A	109.5	H15A—C15—H15B	107.8
N2—C2—H2B	109.5	N4—C16—C15	110.99 (15)
C1—C2—H2B	109.5	N4—C16—H16A	109.4
H2A—C2—H2B	108.1	C15—C16—H16A	109.4
N2—C3—C4	121.28 (17)	N4—C16—H16B	109.4
N2—C3—H3	119.4	C15—C16—H16B	109.4
C4—C3—H3	119.4	H16A—C16—H16B	108.0
C5—S1—C6	91.85 (9)	N4—C17—C18	122.64 (17)
C5—C4—C7	111.84 (16)	N4—C17—H17	118.7
C5—C4—C3	123.20 (17)	C18—C17—H17	118.7
C7—C4—C3	124.89 (15)	C19—S3—C20	90.7 (2)
C4—C5—S1	111.98 (14)	C21—C20—S3	111.6 (4)
C4—C5—H5	124.0	C21—C20—H20	124.2
S1—C5—H5	124.0	S3—C20—H20	124.2
C7—C6—S1	111.50 (14)	C21—S3A—C20A	89.8 (5)
C7—C6—H6	124.3	C19—C20A—S3A	112.0 (8)
S1—C6—H6	124.3	C19—C20A—H20A	124.0
C6—C7—C4	112.84 (16)	S3A—C20A—H20A	124.0
C6—C7—H7	123.6	C19—C18—C21	111.44 (17)
C4—C7—H7	123.6	C19—C18—C17	125.50 (17)
N1—C8—C9	112.52 (15)	C21—C18—C17	123.04 (17)
N1—C8—H8A	109.1	C20A—C19—C18	108.6 (5)
C9—C8—H8A	109.1	C18—C19—S3	113.67 (15)
N1—C8—H8B	109.1	C20A—C19—H19	128.1
C9—C8—H8B	109.1	C18—C19—H19	123.2
H8A—C8—H8B	107.8	S3—C19—H19	123.2
N3—C9—C8	111.31 (15)	C20A—C19—H19A	125.7
N3—C9—H9A	109.4	C18—C19—H19A	125.7
C8—C9—H9A	109.4	S3—C19—H19A	120.6
N3—C9—H9B	109.4	C20—C21—C18	112.6 (3)
C8—C9—H9B	109.4	C18—C21—S3A	118.0 (3)
H9A—C9—H9B	108.0	C20—C21—H21	123.7
N3—C10—C11	122.30 (16)	C18—C21—H21	123.7
N3—C10—H10	118.9	S3A—C21—H21	118.3
C11—C10—H10	118.9	C20—C21—H21A	126.4
C12—C11—C14	111.82 (17)	C18—C21—H21A	121.0
C12—C11—C10	122.64 (16)	S3A—C21—H21A	121.0
			
C8—N1—C1—C2	−80.05 (18)	C10—C11—C14—C13	−175.99 (16)
C15—N1—C1—C2	156.79 (15)	C8—N1—C15—C16	156.43 (15)
C3—N2—C2—C1	109.39 (18)	C1—N1—C15—C16	−80.51 (18)
N1—C1—C2—N2	−74.34 (19)	C17—N4—C16—C15	120.22 (18)
C2—N2—C3—C4	176.83 (15)	N1—C15—C16—N4	−75.56 (19)
N2—C3—C4—C5	−173.54 (17)	C16—N4—C17—C18	177.60 (16)
N2—C3—C4—C7	3.2 (3)	C19—S3—C20—C21	−1.2 (6)
C7—C4—C5—S1	−0.38 (19)	C21—S3A—C20A—C19	3.9 (11)
C3—C4—C5—S1	176.71 (13)	N4—C17—C18—C19	−10.2 (3)
C6—S1—C5—C4	0.30 (15)	N4—C17—C18—C21	171.36 (18)
C5—S1—C6—C7	−0.13 (15)	S3A—C20A—C19—C18	−4.2 (11)
S1—C6—C7—C4	−0.1 (2)	S3A—C20A—C19—S3	146 (7)
C5—C4—C7—C6	0.3 (2)	C21—C18—C19—C20A	2.7 (7)
C3—C4—C7—C6	−176.75 (16)	C17—C18—C19—C20A	−175.9 (7)
C15—N1—C8—C9	−79.14 (17)	C21—C18—C19—S3	−0.5 (2)
C1—N1—C8—C9	157.46 (15)	C17—C18—C19—S3	−179.13 (15)
C10—N3—C9—C8	112.60 (19)	C20—S3—C19—C20A	−30 (6)
N1—C8—C9—N3	−74.04 (19)	C20—S3—C19—C18	1.0 (4)
C9—N3—C10—C11	176.80 (15)	S3—C20—C21—C18	1.2 (8)
N3—C10—C11—C12	−178.59 (18)	S3—C20—C21—S3A	−172 (9)
N3—C10—C11—C14	−2.2 (3)	C19—C18—C21—C20	−0.4 (5)
C14—C11—C12—S2	−0.2 (2)	C17—C18—C21—C20	178.2 (5)
C10—C11—C12—S2	176.66 (13)	C19—C18—C21—S3A	0.3 (5)
C13—S2—C12—C11	−0.32 (15)	C17—C18—C21—S3A	178.9 (5)
C12—S2—C13—C14	0.73 (14)	C20A—S3A—C21—C20	5(8)
S2—C13—C14—C11	−0.95 (19)	C20A—S3A—C21—C18	−2.3 (8)
C12—C11—C14—C13	0.7 (2)		

### Hydrogen-bond geometry (Å, °)

**Table:**

Cg1, Cg2 and Cg3 are the centroids of the S1,C4–C7, S2,C11–C14 and S3,C18–C21 rings, respectively.

**Table:**

*D*—H···*A*	*D*—H	H···*A*	*D*···*A*	*D*—H···*A*
C20—H20···N2^i^	0.95	2.55	3.354 (7)	143
C21—H21···Cg1	0.95	2.61	3.437 (2)	146
C5—H5···Cg2	0.95	2.65	3.452 (2)	142
C12—H12···Cg3	0.95	2.61	3.432 (3)	145

Symmetry codes: (i) *x*, −*y*+1, *z*−1/2.
